# Outcomes and Challenges in the Programmatic Implementation of Tuberculosis Preventive Therapy among Household Contacts of Pulmonary TB Patients: A Mixed-Methods Study from a Rural District of Karnataka, India

**DOI:** 10.3390/tropicalmed8120512

**Published:** 2023-11-30

**Authors:** U. C. Samudyatha, Kathirvel Soundappan, Gomathi Ramaswamy, Kedar Mehta, Chandan Kumar, M. Jagadeesh, B. T. Prasanna Kamath, Neeta Singla, Pruthu Thekkur

**Affiliations:** 1 Department of Community Medicine, Sri Devaraj Urs Medical College, Sri Devaraj Urs Academy of Higher Education and Research (SDUAHER), Tamaka, Kolar 563101, India; btpkamath@gmail.com; 2 Department of Community Medicine and School of Public Health, Postgraduate Institute of Medical Education and Research, Chandigarh 160012, India; selvkathir@gmail.com; 3 Department of Community Medicine and Family Medicine, All India Institute of Medical Sciences, Bibinagar, Hyderabad 508126, India; gmthramaswamy@gmail.com; 4 Department of Community Medicine, GMERS Medical College, Gotri, Vadodara 390021, India; kedar_mehta20@yahoo.co.in; 5 District Health Office, Kolar 536101, India; dtokakol@rntcp.org (C.K.); dhoklr@gmail.com (M.J.); 6 National Institute for Tuberculosis Research and Respiratory Diseases, New Delhi 110030, India; docneetasingla@gmail.com; 7 Centre for Operational Research, International Union against Tuberculosis and Lung Disease, 2 Rue Jean Lantier, 75001 Paris, France; pruthu.tk@theunion.org

**Keywords:** isoniazid preventive therapy, tuberculin skin test, contact management, contact investigation, latent tuberculosis, QuantiFERON-TB Gold test, IFN-γ, operational research, structured operational research and training initiative

## Abstract

The National TB Elimination Programme (NTEP) of India is implementing tuberculosis preventive treatment (TPT) for all household contacts (HHCs) of pulmonary tuberculosis patients (index patients) aged <5 years and those HHCs aged >5 years with TB infection (TBI). We conducted an explanatory mixed-methods study among index patients registered in the Kolar district, Karnataka during April-December 2022, to assess the TPT cascade and explore the early implementation challenges for TPT provision. Of the 301 index patients, contact tracing home visits were made in 247 (82.1%) instances; a major challenge was index patients’ resistance to home visits fearing stigma, especially among those receiving care from the private sector. Of the 838 HHCs, 765 (91.3%) were screened for TB; the challenges included a lack of clarity on HHC definition and the non-availability of HHCs during house visits. Only 400 (57.8%) of the 692 eligible HHCs underwent an IGRA test for TBI; the challenges included a shortage of IGRA testing logistics and the perceived low risk among HHCs. As HHCs were unaware of their IGRA results, a number of HHCs actually eligible for TPT could not be determined. Among the 83 HHCs advised of the TPT, 81 (98%) initiated treatment, of whom 63 (77%) completed treatment. Though TPT initiation and completion rates are appreciable, the NTEP needs to urgently address the challenges in contact identification and IGRA testing.

## 1. Introduction

Tuberculosis (TB) is a global public health problem, as an estimated 10.6 million individuals fell ill and 1.6 million died due to TB globally in 2022. Globally, one out of every two TB patients experiences catastrophic expenditure due to the disease [1]. To overcome the high mortality and catastrophic expenditure due to TB, the World Health Organisation’s (WHO) END TB Strategy has set a target of reducing the incidence of TB disease by 90% between 2015 and 2030 [2].

A key strategy for reducing the incidence of TB disease is to limit individuals from acquiring TB infection (TBI) and prevent those with TBI from developing TB disease. The WHO recommends TB preventive treatment (TPT) to HHCs of bacteriologically confirmed pulmonary TB patients to reduce the risk of developing TB disease among those with TBI [3]. TPT for at-risk populations results in a 10% annual rate of decline in TB incidence [4]. 

During the United Nations High-Level Meeting (UNLHM) on the fight against TB in 2018, world leaders committed to providing TPT to 4 million household contacts aged <5 years and 20 million household contacts aged ≥5 years by 2022 [5]. However, about 2.2 million (55% of the target) household contacts <5 years and only 2 million household contacts aged ≥5 years (10% of the target) received TPT between 2018 and 2022 [1]. A recent meta-analysis (2022) reported that the coverage of household contact screening and TPT varied from 14% to 100% in low- and middle-income countries [6]. Studies from similar settings have reported sub-optimal implementation of contact tracing and TPT due to challenges in the identification of household contacts, poor access to testing for TBI, lack of awareness about TPT among healthcare providers and contacts, inadequate supply of TPT drugs and low prioritisation of TPT in the programme [7,8,9]. Despite these gaps, the recent UNLHM in 2023 highlighted the importance of TPT and committed to providing TPT to 30 million HHCs between 2023 and 2027 [10].

India is a high TB burden country, contributing to more than one-fourth of the estimated global incidence [1]. The National TB Elimination Programme (NTEP) of India is working on a mission mode to eliminate TB by 2025, a decade ahead of the global target [4]. The guidelines on the Programmatic Management of TB Preventive Treatment in India (2021) recommend contact management (identification, screening and investigation) of all the household contacts of pulmonary TB patients (index patients). TPT is recommended for household contacts aged >5 years with TBI and all household contacts aged ≤5 years [4]. Amongst the various options recommended in the WHO guidelines, during the study period, 6 months of daily isoniazid was given as the TPT [11].

Contact tracing and provision of TPT include sequential steps (cascade), starting with a contact tracing visit by a healthcare worker to the house of the TB index patient, screening of the household contacts for symptoms suggestive of TB, contact clinical examination to rule out active TB, testing for TBI, ascertaining TPT eligibility, initiation of TPT and providing treatment adherence support and ascertainment of TPT outcomes [4]. It is important to assess and monitor the performance at each step of the cascade to identify gaps and make informed decisions to improve performance. 

The NTEP monitors the performance at some of the steps of the cascade using NIKSHAY, an electronic patient management system. The indicators in the NIKSHAY are currently deduced using aggregate data and individual-level details of each identified household contact are not available. Thus, the risk groups for attrition at each step of the cascade cannot be identified. Also, the reasons for identified gaps are not available. Thus, there is a need for qualitative exploration among the healthcare workers, TB index patients and their contacts to comprehensively understand the causes of the gaps in the cascade and to find possible solutions to overcome such gaps in this early implementation phase. 

Thus, we conducted a study among drug-susceptible pulmonary TB patients who were initiated on treatment in the Kolar district of Karnataka, India from April to December 2022 and their household contacts to describe the coverage at each step of the TPT cascade. We also explored the perception of the TB index patients, household contacts and healthcare providers on the enablers and challenges in implementing contact tracing and TPT provision. 

## 2. Materials and Methods

### 2.1. Study Design

This was an explanatory mixed-methods study (sequential) with a quantitative cohort study using routinely collected programmatic secondary data updated via telephone calls followed by a qualitative descriptive study.

### 2.2. Study Setting

#### 2.2.1. General Setting

The study was conducted in Kolar district of Karnataka, a south Indian state. The state has a population of about 72 million and an estimated TB disease prevalence of 269.9 (181.7–358.1) per 100,000 population [12]. TB healthcare services are provided through both the public and private sectors in the state. The public sector has a three-tier system and provides services free of cost. The state also has a widespread private healthcare sector where individuals avail services with out-of-pocket expenditure or health insurance. 

#### 2.2.2. Specific Setting 

##### Organisation of TB Care Services in Kolar District

Kolar district is the easternmost district of Karnataka, with a population of about 1.5 million. About two-thirds of the residents live in rural areas while industrial areas have emerged in the past decade. The district is divided into six Tuberculosis Units (TU). In four TUs, screening adult household contacts (HHCs) of bacteriologically confirmed pulmonary TB patients with interferon-gamma release assay (IGRA) for TBI and treating TB-infected HHCs with a 6H regimen (isoniazid for six months) was initiated in January 2022 (Mulabgilu, Srinivaspura, Kolar and Malur). TUs are located at the sub-district level with a Medical Officer—TB Control and designated Senior Treatment Supervisor (STS). There are 267 peripheral health institutions (PHI) under these four TB Units. Sub-centre Health and Wellness Centres are PHIs run by Community Health Officers (CHOs) and Multipurpose Health Workers (male and female) with the assistance of one ASHA (Accredited Social Health Activist) per village. The staff of Primary Health Centres (Medical officer, Auxiliary Nurse Midwife-ANM, Health Assistant, Health Educator, Pharmacist and Lab technician) and those of Sub-Centre Health and Wellness Centres are involved in the diagnosis of TB patients, contact tracing visits, sample collection for IGRA and provision of ATT and TPT. All health facilities (public/private) mandatorily report TB patients detected in their facility through an e-portal, named NIKSHAY. 

##### Diagnosis and Treatment of TB Patients

The individuals with symptoms suggestive of TB are identified and evaluated for TB. Irrespective of the place of diagnosis, the patient is linked to the PHI closest to their residence for further care provision, including contact tracing and TPT provision. 

##### Contact Tracing Visits and Identification of Household Contacts

As per NTEP guidelines, the STS, the TB Health Visitor (TB-HV) or the frontline health workers of the PHI make a contact tracing visit to the houses of all the pulmonary TB patients to list all HHCs and initiate the process of screening HHCs. As per the guidelines, HHC is defined as “a person who shared the same enclosed living space as the index TB patient for one or more nights or for frequent or extended daytime periods the three months before the start of current TB treatment” [4].

##### Screening of Household Contacts

The HHCs are first evaluated for the presence of active TB disease through symptom screening. Symptoms include any one of the following: cough, fever, night sweats, haemoptysis, weight loss, chest pain, shortness of breath or fatigue. In children <5 years, symptoms also include anorexia, failure to thrive, not eating well, decreased activity or playfulness [4]. If found symptomatic, they are considered presumptive TB patients and evaluated for active TB disease (TBD) using sputum examination (CBNAAT) and chest X-ray. Those HHCs with confirmed (microbiological/clinical) TBD are linked for anti-TB treatment at PHI. 

HHCs aged less than five years, with no symptoms of active TB, are provided with TPT for six months. Those HHCs aged ≥5 years are screened for TBI using IGRA. The blood samples are collected by field staff and sent to district hospitals or medical colleges for IGRA tests. Those screened positive for TBI and with a normal chest X-ray are initiated on TPT. If chest X-ray is not available or normal, TPT is initiated after ruling out contraindications like hepatitis, peripheral neuropathy and regular alcohol consumption, regardless of the history of TB. Similarly, HHC has to be started on TPT if the IGRA test is not available after ruling out TBD and contraindications. HHC on TPT is given a TPT card and followed up monthly by respective PHC or HCW staff usually through telephone calls or personally during the monthly dispensation of TPT.

### 2.3. Study Population

#### 2.3.1. Quantitative Component

All the drug-sensitive pulmonary TB patients (TB index patients) on TB treatment in the selected four TUs of Kolar district from April to December 2022 and their HHCs were eligible for the study. Index patients who did not have a valid telephone number and therefore lost to follow-up during treatment or in whom the regimen was changed were excluded from the study. 

#### 2.3.2. Qualitative Component

Purposive sampling method was used to select healthcare workers (HCWs), ensuring participation from all four TUs and different cadres: three STS, three medical officers of PHIs, two TB-HV, one CHO, one health educator and one pharmacist for the in-depth interview. TB index patients (2) and household contacts (6) were approached using extreme variation sampling. All those approached consented to take part in the interview. The total number of interviews was guided by information saturation.

### 2.4. Data Variables and Data Collection

#### 2.4.1. Quantitative Component

Data on the socio-demographic profile (age, gender and TU), and clinical profile (type of diagnosis, previous history of TB treatment and health sector of diagnosis) of the index drug-sensitive pulmonary TB patient were extracted from the TB notification registers of programmatic data. The aggregate number of household contacts (stratified by <5 years and ≥5 years), the number screened for symptoms of TB, the number with symptoms suggestive of TB, the number evaluated and diagnosed with TB and the number initiated on anti-TB treatment and TPT were extracted from the contact tracing register (Supplementary Table S1). The PI obtained the data during the first week of March 2023. 

The PI contacted the TB index patients or their contacts over the phone and interviewed them about the receipt of contact tracing and TPT services. The data were collected in the local language using a structured questionnaire (Supplementary Table S2). Details of each household contact like age, gender, relationship with the index patient, history of screening for TB disease and TB infection, TPT initiation and completion were obtained.

#### 2.4.2. Qualitative Component

All the interviews were conducted by the PI, who is a female medical doctor, fluent in the local language (Kannada) and trained in qualitative research methods. The interviews were conducted using the interview guides (Supplementary Tables S3 and S4) and revised based on the quantitative findings. The interviews were conducted in privacy at the residence of household contact and offices of the healthcare workers. All the interviews were conducted in the local language and were audio-recorded using an audio-recording device. On average, each interview lasted for 45 min. 

### 2.5. Data Entry and Analysis 

#### 2.5.1. Quantitative Component 

TB notification register and contact tracing registers were merged using the Episode ID (a unique identifier) of each TB index patient. Data collected through telephone interviews were entered into the merged Microsoft Excel database. Data were analysed using Stata version 14. Data on contact tracing and TPT cascade collected from programmatic data and through telephone interview data were presented as separate flow charts. The number and proportion of index patients and HHCs in each step of the cascade (contact tracing visit, screening for TB symptoms, evaluating for TB disease, testing for TBI, initiation and completion of TPT) was calculated. Unadjusted binominal regression was used to assess the association of sociodemographic and clinical characteristics of index patients and household contacts with four outcomes, namely, not making contact tracing visits, not screening for TB symptoms, not tested for TBI and Under 5 HHCs not provided TPT. Crude relative risk with 95% CI was used as a measure of association.

#### 2.5.2. Qualitative Component

Transcripts were prepared in the English language by listening to audio records within two days of the interview. Manual thematic analysis was carried out by UCS and PT, to identify themes under the broad topics: enablers, challenges and suggested solutions, from the perspectives of household contacts, index patients and healthcare workers at different levels of the TPT cascade. The analysis was reviewed by a third author (KS) and any disagreements between researchers were resolved by discussion. The final table arising after qualitative data analysis was shared with the stakeholders for their feedback and approval. The findings were reported according to COREQ (Consolidated Criteria for Reporting Qualitative Research) guidelines [12,13].

## 3. Results 

In total, 955 drug-sensitive TB patients were notified in the Kolar district from April to December 2022. Of the total, 732 (77%) were pulmonary TB patients. Of the 732 pulmonary TB patients, 53 were either lost to follow-up or diagnosed with drug-resistant TB and were excluded. Also, 292 pulmonary TB patients from two TUs where TPT had not been implemented were excluded. Thus, we listed 487 pulmonary TB patients (index patients) for enrollment in the study. 

Among the 487 index patients, we were able to interview and include 301 (62%) patients since 154 were not reachable telephonically (wrong number/out of coverage area/did not attend the call) and 29 patients did not consent. Among the 183 index patients (138 men and 45 women) who could not be interviewed, 25 (13.6%) had completed treatment, 34 (18.4%) had died while 124 were on treatment at the time of the study. Of the 301 index patients included, 219 (72.7%) were males, 265 (88%) had bacteriologically confirmed TB, 268 (89%) were newly diagnosed and 289 (96%) were availing treatment from the public sector. 

**Figure 1:**
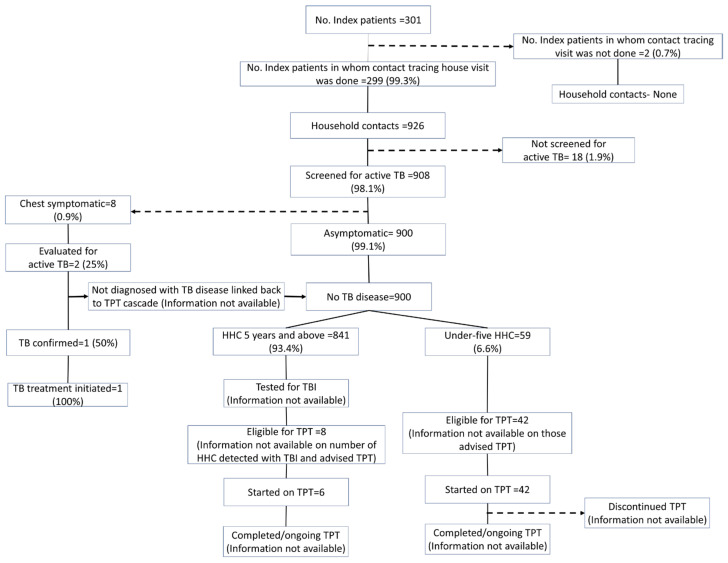
TPT cascade constructed using the routinely collected programmatic data of household contacts of drug-susceptible pulmonary TB patients initiated on anti-TB treatment between April and December 2022 in Kolar district, Karnataka (numbers as entered in the digital platform till the date of extraction).

Using the programmatic data, the contact tracing and TPT cascade of the 301 TB index patients and their contacts is provided in Figure 1.

**Figure 2:**
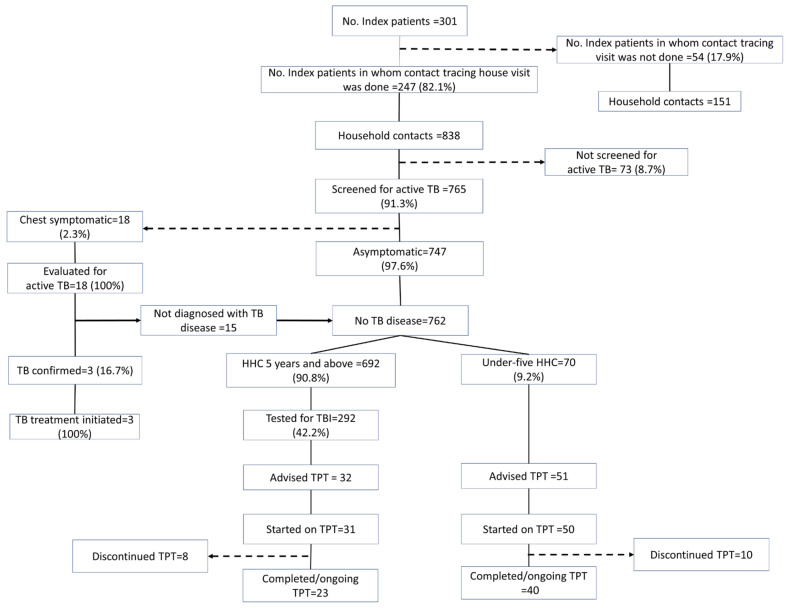
TPT cascade constructed using data collected through telephonic interviews of household contacts and index drug-sensitive pulmonary TB patients initiated on anti-TB treatment between April and December 2022 in Kolar district, Karnataka.

**Figure 3:**
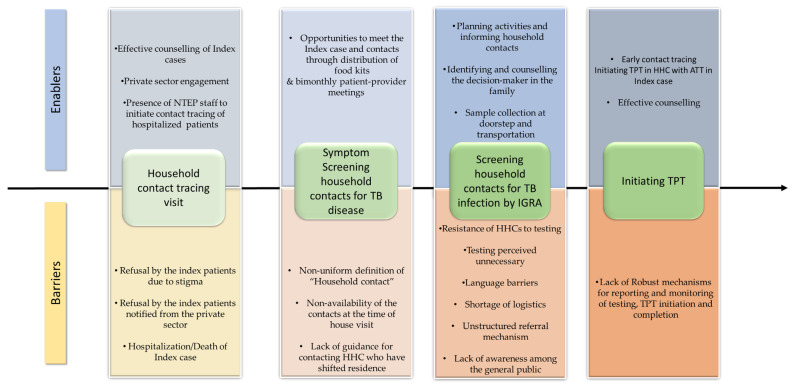
Enablers and barriers at different levels of TPT cascade among household contacts of pulmonary TB index patients who initiated anti-TB treatment between April and December 2022 in Kolar district, Karnataka.

The contact tracing and TPT cascade of the 301 TB index patients and their contacts, based on the interviews, is provided in Figure 2. Enablers and barriers encountered at each step of the TPT cascade, identified in the thematic analysis, are represented in Figure 3 and detailed in the text.

### 3.1. Contact Tracing Visits

According to programme data, contact tracing visits were made to 299 (99%) of the 301 index patients included in the study. As per the interviews, coverage of the household contact tracing was 82.1% (95% CI: 77.2%–86.2%). The sociodemographic and clinical characteristics of the index patients that were associated with no contact tracing visits by the healthcare workers are described in Table 1. The TB index patients of TU-3 were 3.7 times (95% CI 1.4–9.9) more likely not to be visited by the healthcare worker for contact tracing compared to the index patients from TU-4. 

Enablers and barriers encountered at each step of the TPT cascade, identified in thematic analysis, are represented in Figure 3.

#### 3.1.1. Enablers in Conducting Contact Tracing Visits

##### Enabler 1: Effective Counselling of the Index Patients and Assuring Confidentiality

The healthcare workers mentioned that the prior counselling of the index patients about contact tracing helps in conducting the activity. Also, they felt it was necessary to ensure the patients that confidentiality would not be breached during the contact tracing visit.

“*We also assure them that we will not inform other people. Even if they (neighbours) ask, we will tell them that the person has a cough—they think that we have come to give free medicines. But we don’t tell anybody that he has TB. The household contacts have to be informed because they have to be protected*”.

*HCW 3*

HCWs mentioned that the index patients cooperated in providing household contacts after the need for contact tracing to protect the family members was explained. The improved health literacy and awareness about contact tracing due to COVID-19 have been beneficial.

“*So we call them up before going and make sure all of them stay—but some people ask why? We have to make them understand. But the COVID scenario has helped us of late. People have some awareness—they understand what is contact tracing and what to expect. Without COVID, it would have been tough—definitely been tough*”.

*HCW4*

##### Enabler 2: Private Sector Engagement

The HCWs felt that the sensitisation of the private sector was essential in the smooth conduct of the contact tracing visits to the houses of the index patients diagnosed in the private sector. The private providers support the programme staff in carrying out the contact tracing activity.

“*They accept it better when their doctor (private sector) tells them. We meet different private practitioners in the town once fortnightly. We tell them to refer patient contacts for IGRA also. Sometimes they do, but this requires more campaigning and awareness generation*”.

*HCW1*

##### Enabler 3: Visit of NTEP Staff to Private Sector Tertiary Care Hospitals for Initiating Contact Tracing

Though there are no guidelines for contact screening of a hospitalised patient and further follow-up in the field, the NTEP staff in the district identified index patients admitted for a long duration in the health facility and initiated contact tracing.

“*She was admitted to a private hospital for about a month with TB. She was in hospital for about a month. The Government health workers have a counter—some office like that in that hospital. So they came to take her sample for testing. We were taking turns to look after her. So they took our details and asked us to be tested as well*”.

*HHC5*

#### 3.1.2. Challenges in Conducting Contact Tracing Visits

##### Challenge 1: Refusal by the Index Patients due to Stigma

One of the reasons for the refusal by the index patients for contact tracing visits by healthcare workers was due to fear of the stigma about TB and discrimination by the household members and neighbours, post disclosure.

“*It would be difficult if they come here. The family will get disturbed. Small kids are there. They will get scared. All the neighbours will ask. anyway I go and take the medicines there. So they need not come here*”.

*Index patient 2*

In some cases, the index patients hesitated to allow health teams consisting of four-five health care workers to visit the house since it draws the attention of the neighbours/villagers.

“*Some people are cooperative. Some of them do not want so many people to visit their house at a time. They will not be cooperative because they feel that people will stigmatize them. In such case, we ask them to come to the PHC itself and we take history here itself*”.

*HCW 2*

##### Challenge 2: Refusal by the Index Patients Notified from the Private Sector

Healthcare workers also expressed how patients notified from private hospitals were hesitant for contact tracing. There were fewer opportunities to counsel and gain the confidence of the index patients from the private sector for contact tracing.

“*We had a case recently that was notified from a private hospital. They took ATT in a private hospital. They refused to allow us to do a house visit or contact tracing. They did not allow our staff to contact them or explain. When they get treatment from us, we meet them repeatedly. But if they refuse to take treatment from us, it will be difficult for us to trace the contacts*”.

*HCW6*

In some situations, challenges arose when the index patient was better educated than the field HCW. Such index patients refused to be monitored for adherence to treatment or follow-ups, or cooperate for contact tracing. HCWs also narrated instances where contact tracing visits could not be carried out among patients lost to follow-up.

“*The field staff have no problem in going to their [those notified through private sector] house. But they [patient] may be reluctant. Serious problem sometimes—I had a well-educated patient who had TB and was reluctant to take the tablets. We could not do a contact tracing in that house also, because she did not allow it. Then in such cases, my staff also found it difficult—because in cases where it is someone who has lower educational status and understanding capacity than my staff, then they will find it easy—To communicate and to explain in their own language. When a learned person refuses, they find it so difficult*”.

*HCW4*

##### Challenge 3: Hospitalisation/Death of Index Patient

The healthcare workers felt that a delay in the diagnosis of index patients, a delay in reporting by tertiary care hospitals through NIKSHAY and the shifting of critically ill patients from elsewhere without notification delayed contact tracing activities.

“*Actually we did not pick up any calls for a long time [after her death]. Relatives and others were calling. She was young and healthy. suddenly she died. so it was very disturbing. And they kept her [in the hospital] for so many days. We were angry also. So, we did not follow up on anything*”.

*HHC2*

Apart from the non-availability of a correct address or additional contact details, the death of the index patient usually discourages the healthcare worker from approaching the family of the deceased, due to the apprehended non-response from the HHCs.

“*Usually they will not respond. Sometimes address will not be correct, so we may not be able to contact. In some cases, because they have rituals extending up to 15 days [after death], they do not entertain us. Even if we meet them, we might miss out on a few. They are usually agitated by the death of a family member. They are frustrated by the health system also*”.

*HCW1*

### 3.2. Identification of Household Contacts and Evaluation for Active TB

According to the NIKSHAY data, 926 household contacts were identified for 299 index patients, with an average of 3.1 household contacts per index patient. Of the 926 contacts, 908 were screened for symptoms suggestive of TB. Whereas, according to the interviews, 838 household contacts were identified for 247 index patients, with an average of 3.4 household contacts per index patient. Of the 838 identified household contacts, 765 (91.3%, 95% CI: 89.2–93.1) were screened for symptoms suggestive of TB. 

The HHCs were less likely to be screened for symptoms if they were male or were children, vertically extended family members or roommates of the index patient (Table 2).

#### 3.2.1. Enablers for Identification of a “Household Contact” and Screening

Additional opportunities to sensitise the index patients as well their accompanying HHCs to obtain household screening for TB disease presented themselves during the monthly nutrition kit distribution to the TB patients at the PHC as well as the bimonthly patient–provider meetings with the TB patients.

#### 3.2.2. Challenges in Identification of a “Household Contact” and Screening

The healthcare workers felt that the definition they used for the household contact (HHC) listing was not uniform. Also, the healthcare workers who performed the contact tracing activity differed from place to place and perceived the definition of household contact differently. The definition of household contact varies, as mentioned below:

- Any person who lived in the same house as the index patient after the symptom onset;
- Any person who lived in the same house as the index patient after the detection of TB;
- Definition (i) or (ii) and whoever is in regular contact with the patient at home (e.g., relatives, neighbours, common friends) but ‘regularity’ is not specified;
- Definition (i) or (ii) and whoever is in regular contact with the patient at home or workplace but the duration of contact is not specified;
- Definition (iii) and any visitor (time and duration is not specified);
- Definition (i) or (ii) and any person named by the family;
- Contacts considered ‘close’ to the index patient within the family by the HCW/index patient/other family members.

Definition (iii) was the most used, since they felt over-inclusion was better, compared to inadvertent exclusion.

“*Sometimes there will be visitors. In some cases, there will be a joint family, which has divided. They might be staying in separate houses. Our workers will count them all together. In some cases, they take only the old couple as one family and leave the others out. ASHA usually knows the family composition in her village. The definition varies from house to house. They will usually record whoever is named by the family*”.

*HCW1*

Line listing of the HHCs was performed by the ASHA in the most rural areas and Health Assistants in urban areas and then cross-verified by the CHO, ANM or Medical Officer (PHC). However, the visits by the Medical Officer and STS were not conducted regularly in most places and were reserved for instances where the other HCWs faced difficulties in contact tracing or screening. The contact tracing history elicited by the Medical Officer or STS was more detailed, though the definitions were non-uniform.

At least six different variations of questions were asked by HCWs to enlist the household members:

- Who is there in the family?
- Who stays in this house?
- Whom does the patient/do you stay with?
- Who looks after the patient/you?
- Who is there with the patient/you?
- Who is in contact with the patient/you?

These questions were mainly directed towards the index patient, and sometimes to the household contact. The duration of contact or regularity of contact was not specified in most cases. In some instances, the index patient or household contacts did not name certain persons as “household contacts” because of low perceived risk among them and selectively screened certain family members. In some instances, the family members separated the TB index patients immediately after diagnosis to a separate house to “minimize contact and spread”. Similarly, some family members, especially extended family members, moved out of the house after the diagnosis of the index patient in a bid to “escape from TB disease”. In such cases, these individuals were not considered and named as ‘contacts’ by the household members.

“*My brother’s son was staying with us for about 6 months. He came here to study. Occasionally he would help my husband in his work also. If he gets TB then who will look after him? We don’t have enough money also. So we sent him back to the village as soon as my husband was diagnosed*”.

*HHC4*

##### Challenge 2: Non-Availability of the Contacts at the Time of the House Visit

When an HHC was out at work or school, the HCW relied upon the history given by the other family members, and they were included in the contact list. Pregnant and lactating mothers who were staying in their maternal house at the time of the visit, according to some HCWs, were receptive and could be tracked through their nearest health facilities. However, no guidelines are in place regarding who should screen or test these women. Hence, in some cases, telephone calls were made to the pregnant women, while in some instances, the family was informed to get them tested.

Similarly, identifying and contacting the HHC of migrant labourers, in whom contacts consist of a floating population or might shift immediately after diagnosis of index patients did not have any standard guideline.

*“Some 8–10 people stay in one room. We tell them (Factory owner/labour agent) not to change rooms till the treatment is over—but there will be a fear factor. In our case, two contacts immediately shifted out to another place. We could contact only one person over the phone. He said he had no symptoms and never came for a check-up. So we told him to observe for at least 6 weeks*”.

*HCW4*

Contact tracing of migrant labourers who transferred out or were lost to follow-up also could not be monitored using the existing mechanisms.

“*In case of the TB patient, we would have entered details in NIKSHAY. But in the case of HHC, it is a challenge because the data will not be there. We complete the entry with the information that we get in the beginning. Contact tracing in other places might take some time. It might not be done or be missed in data entry*”.

*HCW5*

### 3.3. IGRA Testing among Eligible HHCs

The data on the IGRA testing were not readily available in the programme data. Among 692 HHCs aged above five years, an IGRA test was not carried out in 400 (57.8%, 95% CI: 54.0–61.5) (Table 3). HHCs of female index patients and HHCs of newly diagnosed patients were less likely to undergo the IGRA test. HHCs who were vertically extended family members or roommates were less likely to undergo the IGRA test.

#### 3.3.1. Enablers for Uptake of IGRA Test 

##### Enabler 1: Planning Activities and Informing Household Contacts 

As with the contact screening for symptoms, a major challenge for IGRA testing for TBI among the adult HHCs was the non-availability of the HHC when the HCW visited the household. However, one health facility overcame this by planning the contact tracing beforehand, taking the index patient into their confidence and informing the family of the visit through ASHA. The contact screening visit, symptom screening and blood collection for IGRA were performed in one single visit.

“*When we plan the visit, we plan everything together, including IGRA. We ask all the family members to come to the PHC or we go to their house after asking if everyone is there. We plan and go*”.

*HCW2*

##### Enabler 2: Identifying and Counselling the Decision-Maker in the Family 

The healthcare workers felt that identifying the key decision maker in the family and convincing them about the IGRA test improved the uptake. Also, healthcare workers tried to engage with the decision makers early by initiating contact tracing and listing soon after the diagnosis of the index patient.

“*When we go out to the field, most of the patients know us. So when we tell them to get IGRA, they feel that it is important and for their own good. But if we tell them all the details in the beginning itself—that after testing, if it is positive, you will have to take medicines for 6 months, then there will be resistance. They will not agree to get tested then. In some houses, we have made 3–5 visits just to convince one person*”.

*HCW2*

##### Enabler 3: Sample Collection and Transportation 

Providing IGRA sample collection at home and fast-tracking chest X-ray facilities in sub-district hospital-level settings for these HHCs decreased the time that the HCW and the HCC spent on screening and testing. 

#### 3.3.2. Challenges for Uptake of IGRA Test

##### Challenge 1: Resistance of HHCs for Testing

In some instances, HHCs resisted testing on the first visit. HHCs resisted testing for a variety of reasons, including lack of information regarding the test and fear of being tested positive and having to have treatment. In other cases, the HHC was not available despite prior intimation either due to work or school. The absence of the decision maker/head of the family at the time of the visit also impacts the acceptance of testing among other household contacts.

“*No, we had no symptoms na. so we did not go. The children have school. their father gets off on Sunday. anyway, no one has any problem. we will go if there is any problem. I will discuss it with their father and let you know*”.

*HHC4*

##### Challenge 2: Unavailability of IGRA Sample Collection and Testing Facilities

In this early implementation phase, IGRA sample collection was not carried out at the doorstep in some instances, due to a lack of testing kits/personnel. In such instances, household members were called to the PHC/Taluk hospital for testing. There was no protocol in place for counselling the contacts before testing or referral mechanism for testing. This led to “selected testing” of some HHCs of a household while leaving out the others.

“*My mother and sister are the ones who were taking care of me. So I told them to come with me and get tested. They were giving me food and cleaning my room and all that. No one else was in direct contact*”.

*Index patient 2*

##### Challenge 3: Testing Perceived as Unnecessary 

This perception was rooted in the belief among the HHCs and the index patients that separating the index patients into a separate room or serving them food separately and wearing masks in the house would prevent TB disease. IGRA was misunderstood as a test to “Detect TB disease by Blood test”. The HHCs were unable to distinguish between TB disease and TB infection and hence did not perceive that infection could have already occurred among the HHCs. 

Some HHCs refused to be tested since they did not have symptoms. It was a common observation by different HCWs that IGRA test uptake was better when offered within days of the diagnosis of the index patient before their symptoms were relieved.

“*Testing should be done as soon as possible. When TB is detected, the HHC are usually scared. So they know that it would spread to them also. They would also want to test. So when we meet them at that time, we immediately get the samples also. In 2 days. maximum in a week*”.

*HCW 5*

Fear of stigma, discrimination from neighbours, as well as fear of being diagnosed with TB themselves also prompted HHCs to refrain from testing. This was associated with the fear of having to stop going to work or suffering from the side effects observed in the index patient.

##### Challenge 4: Non-Availability of IGRA Test Facilities or Lab Technicians in the Health Facility When the HHC Visited

“*We went 3–4 times for that. but they kept telling us to come later. The person who takes blood was not there it seems. Actually, we go to get tablets for him monthly right. so we thought we could get it done along with that*”.

*HHC8*

The HHCs preferred being tested at home, rather than having to travel to a health facility or miss work.

“*The Govt. hospital is about 10 km from here. We have to work in the fields also. We can’t go and get tested. If they want, they can come and do the test. We can’t go there*”.

*HHC6*

In some cases, IGRA testing was not advised by the HCW, due to the inconsistency of facilities (testing and lab technicians) during the implementation phase in the district. The HCW suggested the involvement of private or NGO partners for contact tracing and IGRA testing. Similarly, the IGRA test reports (positive/negative) were not given to those who were tested; instead, those detected as having TBI were advised verbally to start on TPT.

“*Right now, NTEP does not have separate staff to do IGRA testing. There is a heavy workload. In some districts, they have given these responsibilities to private hospitals and NGOs. They will go and collect the sample, test and then send the report to us. That is an easy way.*”

*HCW1*

##### Challenge 5: Language Barrier

Among the migrant population from North and North Eastern India, who did not know the local language, the HCW faced language barriers. In such instances, help was sought from the employers or other workers.

“*It is easier to convince if we know the language. In these cases, I have to tell one person, they will understand something and translate it to them. They say something and then we don’t understand. Some of them don’t know Hindi also. Those barriers are there. Once they start taking medicines from us, we manage somehow*”.

*HCW7*

##### Challenge 6: Lack of Awareness among the General Public 

When CHO or the ANM are unable to convince the family to undergo testing, they seek the help of the STS or a Medical Officer. However, in some cases, counselling sessions are long and repeated. The non-availability of reliable information among the general public was also perceived as a barrier.

“*We do it individually for the ones who do not agree to IGRA. Others, we don’t burden them with too much information. It takes almost half an hour to convince them. If they don’t agree, then it will extend to the next day. We also take the help of the medical officer if required*”.

*HCW*

HCWs suggested that awareness generation among the general public regarding TB disease and infection was also necessary to increase the acceptability of testing.

“*Counseling and awareness generation. It is very difficult to convince people to undertake treatment if awareness is not there. We can’t put that effort every time—it is not possible to put the same amount of effort into every patient every time. When we started, we did it in a mission mode, with a focus only on that. We did not do anything else. But if we do only that and nothing else, then we will not be able to do anything else*”.

*HCW1*

### 3.4. Initiation and Completion of TPT

According to the programme data, 8/841 (<1%) household contacts aged >5 years and 42/59 (71.2%) aged <5 years were labelled as eligible for TPT. Among those eligible, almost everyone (>95%) was initiated on TPT. No data on the receipt of the IGRA test and test results were available. In the interview, the household contacts aged >5 years who underwent IGRA testing were not aware of their test results. According to the interview, only 32/692 (4.6%) household contacts aged >5 years and 51/70 (72.8%) households aged less than 5 years were advised to undergo TPT. Almost all the individuals who had been advised to undergo TPT initiated TPT. The completion rate was more than 75% among those initiated on TPT. 

According to the healthcare workers, initiating TPT among children was more acceptable to people because it was perceived as “necessary” by them.

“*Among children, they accept, because when one person is positive for TB, there is a fear factor in the family–regardless of whether it is pulmonary or extrapulmonary. They are more worried about children than old people–because they are their future*”.

*HCW4*

Early contact tracing helped in ruling out active TB in a child, as well as initiating TPT in a child simultaneously with ATT in the index patient. This also facilitated the procurement of TPT along with ATT for the index patient. 

Counselling using anecdotes/examples that relate to the patient and HHC helped, such as:

“*Counseling is important. We base it on their experience and work. We also give examples-when your kids go for job selection in the police or military 10 years down the lane, they have to have good lungs. They will check their endurance and chest circumference. So if you protect the child now, they will have a better future. Otherwise-Upiritittulu bagalekunte inkim chesidi (If lungs are not good, then what good is it?)*”.

*HCW 2*

### 3.5. Reporting and Monitoring of Testing, TPT Initiation and Completion

The HCW commented on how better recording and monitoring systems were required for contact tracing, IGRA testing and TPT.

“*We don’t report IGRA negative and other household contact details in Nikshay. Actually, that also has to be entered. We have a facility for that also in Nikshay. Numbers, whether they are positive or negative–numbers are entered. However, the names and other details are not entered. That system must be initiated*”.

*HCW 9*

“*Contact tracing details are entered in their (field level HCW) books. This should be done in Nikshay at the PHC level itself. We will be able to monitor what is the coverage and cross-check also. Then it will work. This will help in monitoring also*”.

*HCW2*

ASHA, during her routine visits to the village, enquires whether HHCs are adhering to TPT or not. There are no uniform guidelines on when the visits should be conducted, if HCWs need to be involved in monitoring visits or how treatment adherence is ensured.

“*The important thing is that we have to treat TB infection patients with the same seriousness that we treat TB disease. We have to follow up with them just like that. Then only it will be a success. If we just think that they will come on their own and leave it to them to take the medicines, then they will not do it. We do not have any means to monitor them also. The card is there. For 3 HP something like 99DOTS can be used for follow-up*”.

*HCW1*

## 4. Discussion

This is the first study from South India assessing the outcomes and early implementation challenges of contact tracing and TPT provision through the NTEP, after the launch of TPT for all household contacts. The study has five key findings. First, a contact tracing visit was not carried out in one out of five index patients. Second, about one in ten of the contacts identified were not screened for symptoms suggestive of TB and the percentage of contacts with presumptive TB was low. Third, the IGRA test was performed only in two out of five individuals eligible for testing and the test results (positive/negative) were not communicated to the HHC. Fourth, with the existing recording system, the proportion of HHCs eligible to receive TPT could not be determined. Fifth, there was a high uptake of TPT and a high completion rate among those who were eligible/advised to undergo TPT.

The strengths of this study were that, firstly, since routine programmatic data was supplemented by the individual-level data collected through interviews, we could reflect better on the on-field realities in the early phase of TPT implementation. Second, the use of a mixed-methods design provided better insights into the reasons for the identified gaps in TPT cascades, early implementation challenges, enablers and possible solutions from both patients and providers. Finally, we complied with the STROBE and COREQ guidelines for reporting observational and qualitative studies [13,14]. 

The study has some limitations. First, the index patients who were lost to follow-up during treatment or did not have a valid telephone number were excluded from the study. Among the eligible TB index patients listed to be contacted, since only about 61% could be contacted over the phone and included in the study, selection bias cannot be ruled out. Those included were those on treatment or who completed treatment and it was highly likely that their HHCs were traced and evaluated. Thus, it could have led to an underestimation of attritions in the TPT cascade. Second, either the index patient or one HHC from each home was telephonically interviewed to collect information regarding the entire household. This might have led to information bias, recall bias and misclassification of the receipt of services. Due diligence was adopted during the interviews to ensure these informants were reliable. It was ensured that informants were familiar with services availed by all the HHCs and that consistency in information was checked by repeating the questions. The direction of the impact of this limitation on the TPT cascade estimates cannot be ascertained. Third, the sample size for qualitative interviews was guided by the overall saturation, and not by saturation at each cadre of HCW or category of HHC. However, care was taken to involve HCWs and HHCs from all four TB units. Finally, the study results should be generalized with caution as the practice of contact tracing for HHCs and TPT is context-specific and varies across the districts. Some districts are implementing a ‘Treat all’ policy, which does not require IGRA testing to ascertain eligibility for TPT. Thus, repeat studies in different study settings would be beneficial for generating context-specific evidence around HHC contact tracing and TPT.

In 2018, it was anticipated that the TPT rollout among HHCs in India would be difficult due to challenges in each step of HHC management. The anticipated challenges included a large number of index patients and thereby their contacts, a lack of systems for identifying these contacts, a lack of diagnostic tools for identification of those with TBI and poor acceptance and adherence to TPT among the contacts [15]. In this study, we explored the gaps and challenges in each step of HHC management.

There were gaps in the tracing of the HHCs of the index patients, with contact tracing performed for only about 80% of the index patients. This gap was attributed to refusals by the index patients for contact tracing home visits due to fear of stigma and discrimination from family members and neighbours. HCWs tried to overcome this barrier by counselling the index patient regarding confidentiality and the process of contact tracing, symptom screening and the need for TPT for their contacts. Although HCWs were able to counsel index patients from the public sector, the time and opportunities to meet and counsel the patients notified from the private sector were few. This is despite a well-established private sector engagement in the management of TB disease in India [16]. One of the anticipated challenges expressed in the ALTER expert panel meeting was the “reluctance of clinicians” to provide TPT as well as the lack of awareness among them [17]. Thus, the NTEP programme should convince and garner support from private providers for the management of the HHCs of index patients notified by the private sector. 

Even upon meeting the index patients for contact tracing, the HCWs had challenges in listing the HHCs. This was largely due to a lack of clarity on the definition of “household contact”. The HHC definitions were used and phrased in different ways, by different HCWs, in different contexts. A lack of clarity on the HHC definition can lead to ineffective contact tracing [18]. This lack of clarity contributes to either overestimation or underestimation of the number of HHCs in complex social settings. Thus, the NTEP should provide a structured operational definition of a “household contact” of a TB patient with clarity on the nature, duration and frequency of contact. 

To effectively identify and screen all HHCs, the HCWs had to counsel the index patients and their HHCs. However, some of the HCWs were not trained and were not confident in counselling the index patients and their HHCs. This contributed to high attrition in the identification and screening of HHCs in the study cohort. Thus, there is an urgent need for improving counselling skills among HCWs involved in HHC management. The training manuals for TPT implementation should provide direction on the HCW responsible for counselling for both public and private sectors, and the components of the counselling, such as the need and process of contact tracing.

Pre-visit planning, counselling of the index patient and coordination by the medical officers of respective primary health centres helped in complete contact tracing and screening of all the household contacts for TB symptoms. Planning contact visits during weekends or holidays might help in improving the coverage but are less likely to be feasible with the available staff and facilities in the study setting [6,19]. In a study with a high HIV–TB burden, active TB case finding among the HHCs was found to have higher case detection compared to passive case finding [20]. Similarly, structured contact tracing activities may also identify more HHCs with TB infection [21]. Further, identification and evaluation of TB among contacts who have shifted residence (including pregnant women and migrants) requires the effort and coordination of HCWs in two or more geographical locations. The lack of a clear role definition for cadres of HCWs involved in the TPT cascade can result in implementation challenges [18]. Thus, the NTEP must additionally detail the process in which HCWs must be involved in HHC management, reporting and monitoring and addressing the communication gaps.

Discrepancies were found between the data recorded in the NIKSHAY and the data collected through patient interviews. The programme officers attributed these discrepancies to the delays in updating the data in NIKSHAY. Sometimes, though the HHC tracing was completed on time, the information on the HHC tracing was uploaded on the NIKSHAY portal only at the time of ascertaining the treatment outcomes of the index patients. Although there is a provision for documenting the demographic and investigation details of each HHC on the NIKSHAY portal, it largely remains incomplete. Thus, the current recording format does not allow the calculation of the percentage eligible for TPT and the completion rate.

Perceived low risk of TB infection among household contacts, misconceptions regarding “TB Disease and TB Infection”, fear of stigma and discrimination and fear of side effects of TPT stemmed from the lack of knowledge regarding TB prevention among the general public. This increases the burden on the HCW to convince and counsel the HHCs repeatedly regarding TPT. This warrants the need to spread awareness among the general public, through mass media and inter-sectoral approaches [22].

The major attrition was seen at the step of IGRA testing for detecting TBI. This was largely due to the non-availability of testing services, sub-optimal referral mechanisms and protocols for sample collection and transportation. Though the HCWs had persisted in convincing HHCs to complete IGRA testing, health system-related issues contributed to poor coverage of IGRA testing. Inadequate availability of diagnostics is a challenge reported across studies from various study settings [22]. These issues require administrative-level attention and action.

Additionally, in this study, we identified that household contact tracing activities were most acceptable when it was carried out within days of index patient detection, and less so after symptom resolution in the index patients. This necessitates timeliness metrics for monitoring the timeliness of household contact tracing in TB index patients.

## 5. Conclusions

In conclusion, though TPT initiation and completion rates are appreciable, early implementation challenges exist in contact identification and IGRA testing. The programme needs to urgently address challenges and improve the TPT services. The study brought about four aspects where the programmatic management of tuberculosis preventive therapy must be strengthened in the early phase of implementation and sustained thereafter. Firstly, operational guidelines on HHC management, including counselling the index patient and the HHCs for contact tracing visits, IGRA testing and TPT initiation. The HCWs from both the public and private sectors must be trained at frequent intervals on these guidelines. Secondly, the health system must be strengthened to improve access to IGRA and chest X-rays at a fast-tracked pace. Thirdly, the documentation of HHC management has to be strengthened with individual-level data at each step of the cascade. The provisions must be made for such documentation in the NIKSHAY, to facilitate real-time monitoring and actions. Fourthly, consistent, intersectoral approaches must be adopted to spread awareness regarding Tuberculosis and TPT among healthcare personnel as well as the general public.

## Figures and Tables

**Table 1:** Characteristics of pulmonary TB index patients initiated on anti-TB treatment in selected tuberculosis units of Kolar district during April-December 2022 patients with households not visited by healthcare workers for contact tracing.

Characteristics of Index PTB Patients	Total	Not Visited for Contact Tracing		Crude RR (95% CI) ^2^	*p*-Value
**Total**	n	n	(%) ^1^		
	301	54	(17.9)	-	-
**Age (in years)**					
≤ 14	2	0	(0)	-	
15–29	54	5	(9.3)	Ref	
30–44	77	15	(19.5)	2.1 (0.8–5.4)	0.125
45–59	93	18	(19.4)	2.1 (0.8–5.3)	0.121
≥60	75	16	(21.3)	2.3 (0.9–5.9)	0.082
**Gender**					
Male	219	38	(17.4)	Ref	
Female	80	14	(17.5)	1.0 (0.6–1.8)	0.976
Others	2	2	(100)	-	
**Type of Diagnosis**					
Bacteriologically confirmed	265	47	(17.7)	Ref	
Clinically diagnosed	36	7	(19.4)	1.1 (0.5–2.2)	0.801
**Type of Patient Based on Previous History of TB Treatment**					
New	268	51	(19.0)	2.1 (0.7–6.3)	0.191
Retreatment	33	3	(9.1)	Ref	
**Tuberculosis Unit (TU No.)**					
1	80	13	(16.3)	2.1 (0.8–6.4)	0.149
2	64	9	(14.1)	1.9 (0.6–5.8)	0.262
3	103	28	(27.2)	3.7 (1.4–9.9) *	0.010
4	54	4	(7.4)	Ref	
**Health Facility Sector**					
Public	289	50	(17.3)	Ref	
Private	12	4	(33.3)	1.9 (0.8–4.5)	0.125

^1^ Row percentage among the total number of PTB index patients initiated on anti-TB treatment in each category; ^2^ relative risk (RR) with 95% confidence interval (CI) for not making a contact tracing visit; PTB = pulmonary tuberculosis. * significant at 95% Confidence level.

**Table 2:** Characteristics of TB index patients initiated on anti-tuberculosis treatment in selected tuberculosis units of Kolar district (April to December 2022) and their household contacts, associated with omission from TB symptom screening of household contacts.

Characteristics	Total	HHCs Omitted from Symptom Screening		Crude RR (95% CI) ^2^	*p*-Value
**Total**	n	n	(%) ^1^		
	838	73	(8.7)	-	-
***Characteristics of TB Index patients***					
**Age (in years)**					
≤14	5	0	(0)	-	
15–29	179	16	(8.9)	Ref	
30–44	207	15	(7.3)	0.8 (0.4–1.6)	0.543
45–59	237	23	(9.7)	1.1 (0.6–2.0)	0.791
≥60	210	19	(9.1)	1.1 (0.5–1.9)	0.970
**Gender**					
Male	582	37	(6.4)	Ref	
Female	256	36	(14.1)	2.2 (1.4–3.4) *	<0.001
**Type of Diagnosis**					
Bacteriologically confirmed	746	65	(8.7)	Ref	
Clinically diagnosed	92	8	(8.7)	1.0 (0.5–2.0)	0.996
**Type of Case (Based on** **Previous Treatment History)**					
New	738	63	(8.5)	Ref	
Retreatment	100	10	(10.0)	1.2 (0.6–2.2)	0.625
**Tuberculosis Unit**					
1	233	19	(8.1)	1.2 (0.6–2.4)	0.614
2	176	12	(6.8)	Ref	
3	236	21	(8.9)	1.3 (0.7–2.6)	0.444
4	193	21	(10.9)	1.6 (0.8–3.1)	0.177
**Health Facility Sector**					
Public	807	68	(8.4)	Ref	
Private	31	5	(16.1)	1.9 (0.8–4.4)	0.127
***Characteristics of HHC***					
**Age (in years)**					
≤ 5	79	9	(11.4)	1.4 (0.7–2.6)	0.370
More than 5	759	64	(8.4)	Ref	
**Gender**					
Male	375	42	(11.2)	1.7 (1.1–2.6)	0.023
Female	463	31	(6.7)	Ref	
**Relationship With the Index Patient**					
Spouse/Partner	175	6	(3.4)	Ref	
Child ^3^	259	21	(8.1)	2.3 (1.0–5.7)	0.057
Parent	104	5	(4.8)	1.4 (0.4–4.4)	0.568
Sibling	52	2	(3.9)	1.1 (0.2–5.4)	0.886
Others ^4^	248	39	(15.7)	4.6 (2.0–10.6)	<0.001

^1^ In “Characteristics of Index TB patients”, row percentage among the total number of PTB index patients initiated on anti-TB treatment in each category; In “Characteristics of HHC”, row percentage among the total number of HHCs in each category. ^2^ The relative risk (RR) with 95% confidence interval (CI) for omission from TB symptom screening. PTB = pulmonary tuberculosis. ^3^ Includes children of all age groups. ^4^ Others—includes vertically extended family members and roommates. * significant at 95% Confidence level.

**Table 3:** Characteristics of household contacts (above 5 years) and PTB index patients initiated on anti-tuberculosis treatment in selected tuberculosis units of Kolar district (April to December 2022), associated with eligible HHCs not undergoing IGRA test for TB infection.

Characteristics	Total	IGRA Test Not Performed		Crude RR (95% CI) ^2^	*p*-Value
**Total**	n	n	(%) ^1^		
	692	400	(57.8)		
***Characteristics of TB Index Patients***					
**Age (in years)**					
≤14	5	0	(0)	-	
15–29	142	78	(54.9)	1.2 (0.9–1.4)	0.166
30–44	176	83	(47.2)	Ref	
45–59	197	120	(60.9)	1.3 (1.1–1.6)	<0.001
≥60	172	119	(69.2)	1.5 (1.2–1.8)	<0.001
**Gender**					
Male	496	276	(55.7)	Ref	
Female	196	124	(63.3)	1.1 (1.0–1.3)	0.058
**Type of Diagnosis**					
Bacteriologically confirmed	616	352	(57.1)	Ref	
Clinically diagnosed	76	48	(63.2)	1.1 (0.9–1.3)	0.289
**Type of Case**					
New	614	372	(60.6)	1.7 (1.2–2.3)	0.001
Retreatment	78	28	(35.9)	Ref	
**Tuberculosis Unit**					
1	193	118	(61.1)	1.2 (1.0–1.4)	0.080
2	148	86	(58.1)	1.1 (0.9–1.4)	0.257
3	196	116	(59.2)	1.1 (0.9–1.4)	0.162
4	155	80	(51.6)	Ref	
**Health Facility Sector**					
Public	667	384	(57.6)	Ref	
Private	25	16	(64.0)	1.1 (0.8–1.5)	0.491
***Characteristics of HHC***					
**Gender**					
Male	297	169	(56.9)	Ref	
Female	395	231	(58.5)	1.0 (0.9–1.2)	0.678
**Relationship with the Index Patient**					
Child ^3^	197	106	(53.8)	Ref	
Spouse/Partner	166	94	(56.6)	1.1 (0.9–1.3)	0.590
Parent	99	56	(56.6)	1.1 (0.8–1.3)	0.650
Sibling	50	28	(56.0)	1.0 (0.8–1.4)	0.778
Others ^4^	180	116	(64.4)	1.2 (1.0–1.4)	0.036
**Symptom Screening Status**					
Asymptomatic	678	392	(57.8)	1.0 (0.6–1.6)	0.960
Chest symptomatic ^5^	14	8	(57.1)	Ref	

^1^ In “Characteristics of Index TB patients”, row percentage among the total number of PTB index patients initiated on anti-TB treatment in each category. In “Characteristics of HHC”, row percentage among the total number of eligible HHCs in each category. ^2^ The relative risk (RR) with 95% confidence interval (CI) for not undergoing IGRA testing. PTB = pulmonary tuberculosis. ^3^ Includes children of all age groups. ^4^ Others—includes vertically extended family members and roommates. ^5^ Chest symptomatic, in whom active TB was ruled out.

## Data Availability

Restrictions apply to the availability of these data. Data was obtained from the NTEP programme and supplemented with interviews and are available with the permission of NTEP, India.

## Supplementary Materials

The following supporting information can be downloaded at: https://www.mdpi.com/article/10.3390/tropicalmed8120512/s1, Table S1: Data collection form (Programme data), Table S2: Data collection form (telephone calls), Table S3: In-depth interview guide (tentative) for index case/HHC; Table S4: Key informant interview guide (tentative) to be used with healthcare providers.
