# Supplementary material for: Outcomes and Challenges in the Programmatic Implementation of Tuberculosis Preventive Therapy among Household Contacts of Pulmonary TB Patients: A Mixed-Methods Study from a Rural District of Karnataka, India
Source: Trop Med Infect Dis. 2023 Nov 30;8(12):512. doi: 10.3390/tropicalmed8120512 (PMC10748199; doi:10.3390/tropicalmed8120512)
Supplement: Supplementary file 1 [file tropicalmed-08-00512-s001.zip › tropicalmed-2709792-supplementary.pdf]

Tropical Medicine and Infectious Disease

MDPI

Table S1. Data collection form (Programme data).

Variable	Possible values
NIKSHAY ID	
TB Unit	
Name of case	
Gender of case	1: Male
	2: Female
	3: TG
	9: Not recorded
Age of case	1 to 100,999:
Mobile number of the patient	
Type of case	1: New
	2: Recurrent
	3: Transferred in
	4: Treatment after failure
	5: Treatment after loss to follow up
	6: Other previously treated
	9: Not recorded
No. of U5 household contacts	
No. of U5 household contacts screened	
No. of U5 household contacts with TB symptoms	
No. of U5 household contacts evaluated	
No. of U5 household contacts diagnosed with TB	
No. of U5 household contacts put on TB treatment	
No. of U5 household contacts eligible for TPT	
No. of U5 household contacts initiated on TPT	
No. of Other household contacts	
No. of Other household contacts screened	
No. of Other household contacts with TB symptoms	
No. of Other household contacts evaluated	
No. of Other household contacts diagnosed with TB	
No. of Other household contacts put on TB treatment	
No. of Other household contacts eligible for TPT	
No. of Other household contacts initiated on TPT	

Table S2. Data collection form (telephone calls).

Variable	Possible values
NIKSHAY ID of index case	
Name of the index case	
Name of HHC	
Was the HHC a part of the household at the time of detection of index case?	0:No
	1:Yes
	9:Do not recall
Age of HHC	0 to 100, 999:
Sex of HHC	1:Male
	2:Female

	3:TG
Was the HHC severely ill/hospitalised at the time of detection of index case?	0:No
	1:Yes
	8:Not applicable
	9:Do not recall
Was the HHC pregnant/lactating at the time of detection of index case?	0:No
	1:Yes
	8:Not applicable
	9:Do not recall
Did HHC have cough/fever/poor weight gain at the time of detection of index case?	0:No
	1:Yes
	8:Not applicable
	9:Do not recall
Was the HHC screened for these symptoms by an HCW?	0:No
	1:Yes
	8:Not applicable
	9:Do not recall
Why was the HHC not screened?	9:Not applicable
Was a diagnosis of active TB made after screening?	0:No
	1:Yes
	8:Not applicable
	9:Do not recall
Was TB treatment initiated?	0:No
	1:Yes
	8:Not applicable
	9:Do not recall
Why was the TB treatment not initiated?	9=Not applicable
What is the status of TB treatment?	1:Completed
	2:Ongoing
	3:Discontinued
	4:Loss to follow up
	5:Died
	6:Failure
	7:Other
	8:Not applicable
	9:Do not recall
Why was TB treatment not completed?	9=Not applicable
Was blood test taken?	0:No
	1:Yes
	8:Not applicable
	9:Do not recall
Was chest X-ray taken?	0:No
	1:Yes
	8:Not applicable
	9:Do not recall
Was TPT advised?	0:No
	1:Yes
	8:Not applicable
	9:Do not recall

Was TPT initiated?	0:No
	1:Yes
	8:Not applicable
	9:Do not recall
Why was the TPT not initiated?	:9=Not applicable
What is the current status of TPT?	1:Completed
	2:Ongoing
	3:Discontinued
	4:Loss to follow up
	5:Died
	6:Failure
	7:Other
	8:Not applicable
	9:Do not recall
Why was TPT not completed?	

Table S3. In-depth interview guide for index case/HHC.

Participant: Case/HHC	
Current status of TB treatment/ TPT:	
Age of the participant:	Gender of the participant:
Date of interview	Name of the interviewer:
After a brief introduction of the project describing its purpose, written informed consent will be sought from the participants to participate in the study. Consent will also be sought to audio record the interview.	
• Can you describe the events that led up to the diagnosis of TB? (Probe–symptoms, hospitalisations, doctor visits, household contacts involved in care).<br>• How was the family informed about the diagnosis of TB? (Probe–who informed, what was the reaction, what were the initial queries made, who addressed the queries, number of health worker contacts, who were there in the household).<br>• Can you describe how the family members were tested for TB? (Probe–who checked, where and when, what was explained to them before the procedure, was assistance of index case taken at any step).<br>• Were all HHCs screened and tested? (Probe–why/why not, any attempt later by HHC/HCW to complete screening, what was told after screening, what was understood by you).<br>• What are the uses of taking TPT? (Probe–source of information, perceived risk of TB).<br>• What do you think the problems in taking TPT are? (Probe–what is the source of the information, was any attempt to clarify the doubts made, how did the HCW respond to your decision).<br>• Why did you decide to take/not take TPT? (Probe–attempts of self-initiated actions to continue on TPT, where did you procure TPT from, ease or difficulty of doing so).<br>• What could have been done to improve the delivery of TPT to you/your household members? (Probe–delivery, enrolment, testing, interaction with health worker).<br>The interview will end after thanking the participant for their time and participation.	

Table S4. Key Informant Interview guide used for healthcare providers.

From the healthcare worker, data will be collected regarding:	
Years of experience of the participant in the NTEP (earlier RNTCP):	
Designation: Field level/supervisory level	
Age of the participant:	Gender of the participant:
Date of interview:	Name of the interviewer:
After a brief introduction of the project describing its purpose, written informed consent will be sought from the participants to participate in the study. Consent will also be sought to audio record the interview.	

- Can you describe the steps that you will take when a patient is diagnosed with TB?
- How effective, is TPT according to you in preventing TB disease?
- How do you plan the contact tracing? (who goes, what is the point of contact, how many times they may have to go, logistics availability)
- What are the challenges that you face when you go for a house visit for screening? (Probe–experiences, hard to reach, resistant communities, unavailability of the contact, transport of samples)
- What are the usual responses of household members when they are told that someone in the family has TB?
- How do you explain TB disease to them?
- How do you explain TPT to household contacts?
- What are the responses of household contacts when informed regarding TPT?
- What are the common reasons for not initiating and discontinuation of TPT? (Probe–experiences)
- What do you do when you have some household contact who is refusing to be screened?
- What do you do when you have some household contact who is refusing to take the TPT? (probe–pregnant, lactating, children, old age)
- When did you train/receive training for TPT? Did you find any challenge in the field that was not earlier explained to you in the training? Can you describe them in detail?
- How can the system be improved to help you better cover TPT beneficiaries? (Probe–digital systems, counselling sessions, drug dose and composition, what will be the time and training required to adopt the solutions?)

The interview will end after thanking the participant for their time and participation.
